# “The Good, the Bad and the Double-Sword” Effects of Microplastics and Their Organic Additives in Marine Bacteria

**DOI:** 10.3389/fmicb.2020.581118

**Published:** 2021-01-20

**Authors:** Víctor Fernández-Juárez, Xabier López-Alforja, Aida Frank-Comas, Pedro Echeveste, Antoni Bennasar-Figueras, Guillem Ramis-Munar, Rosa María Gomila, Nona S. R. Agawin

**Affiliations:** ^1^Marine Ecology and Systematics (MarES), Department of Biology, University of the Balearic Islands, Palma de Mallorca, Spain; ^2^Instituto de Ciencias Naturales Alexander von Humboldt, Universidad de Antofagasta, Antofagasta, Chile; ^3^Grup de Recerca en Microbiologia, Departament de Biologia, Universitat de les Illes Balears, Palma de Mallorca, Spain; ^4^Celomic Unit of the University Institute of Research in Health Sciences of the Balearic Islands, Palma de Mallorca, Spain; ^5^Servicio Científico-Técnicos, University of the Balearic Islands, Palma de Mallorca, Spain

**Keywords:** microplastics, organic additives, marine pollution, cyanobacteria and heterotrophic bacteria, N2-fixing bacteria

## Abstract

Little is known about the direct effects of microplastics (MPs) and their organic additives on marine bacteria, considering their role in the nutrient cycles, e.g., N-cycles through the N_2_-fixation, or in the microbial food web. To fill this gap of knowledge, we exposed marine bacteria, specifically diazotrophs, to pure MPs which differ in physical properties (e.g., density, hydrophobicity, and/or size), namely, polyethylene, polypropylene, polyvinyl chloride and polystyrene, and to their most abundant associated organic additives (e.g., fluoranthene, 1,2,5,6,9,10-hexabromocyclododecane and dioctyl-phthalate). Growth, protein overproduction, direct physical interactions between MPs and bacteria, phosphorus acquisition mechanisms and/or N_2_-fixation rates were evaluated. Cyanobacteria were positively affected by environmental and high concentrations of MPs, as opposed to heterotrophic strains, that were only positively affected with high concentrations of ~120 μm-size MPs (detecting the overproduction of proteins related to plastic degradation and C-transport), and negatively affected by 1 μm-size PS beads. Generally, the organic additives had a deleterious effect in both autotrophic and heterotrophic bacteria and the magnitude of the effect is suggested to be dependent on bacterial size. Our results show species-specific responses of the autotrophic and heterotrophic bacteria tested and the responses (beneficial: the “good,” deleterious: the “bad” and/or both: the “double-sword”) were dependent on the type and concentration of MPs and additives. This suggests the need to determine the threshold levels of MPs and additives concentrations starting from which significant effects can be observed for key microbial populations in marine systems, and these data are necessary for effective environmental quality control management.

## Introduction

Marine coastal ecosystems are the most impacted zones by the pollution of plastics. Up to 10 million tons of plastic enter annually in the oceans (Almroth and Eggert, 2019). This oceanic “soup” of plastic is composed of different particle sizes: macroplastics (>250 mm), mesoplastics (1–25 mm), microplastics (MPs) (1–1,000 μm), and nanoplastics (NPs) (<1 μm) (Hartmann et al., 2019). The most abundant polymers (at the macro- and micro-size scale) on the surface of the oceans and seas are polyethylene (PE), followed by polypropylene (PP) and then by others such as polyvinyl chloride (PVC) or polystyrene (PS) (Suaria et al., 2016). MPs have associated chemical additives (usually organic) that have been added to them to improve their chemical properties, and these low molecular weight additives can leach from the plastic polymers, being also sorbed onto them (Bakir et al., 2014). Therefore, MPs can also be sources and vectors for these organic pollutants, which are deleterious for marine organisms (Hahladakis et al., 2018).

The abundance of plastic particles declines exponentially with depth according to their densities, resulting in low-density polymers (e.g., PE and PP) predominating in the surface waters and higher density polymers (e.g., PS) in the deeper areas (Erni-Cassola et al., 2019). However, some evidence suggests that much of the small plastic particles at the surface, independently of their density, end up in sediments by transport mechanisms (Reisser et al., 2015; Urbanek et al., 2018). The accumulation of MPs at depth indicates the susceptibility of planktonic and benthic macro- and micro-organisms to the effects of these pollutants.

In eukaryotic microorganisms, the deleterious effects of plastics have already been described (Wang and Zheng, 2008; Cole and Galloway, 2015; Nelms et al., 2018). However, studies investigating the direct effects of plastics, especially MPs, on marine prokaryotic organisms (e.g., in their growth, biochemistry or nutrient acquisition mechanisms) are still scarce (Harrison et al., 2011; Bryant et al., 2016; Romera-Castillo et al., 2018; Tetu et al., 2019; Machado et al., 2020; Piccardo et al., 2020; Sarker et al., 2020; Seeley et al., 2020). None have investigated the direct effect of MPs, for example, on marine diazotrophs, which are capable of converting the nitrogen gas (N_2_) into ammonia (NH_3_) through the nitrogenase enzyme complex and playing an important role in the marine N cycle. The few studies that have been done on other microorganisms usually are focused on plastic degradation and biofilm formation (Urbanek et al., 2018). Nonetheless, Machado et al. (2020) and Seeley et al. (2020) suggest that MPs can be anthropogenic stressors affecting microbial diversity and N-cycles. Other studies have reported changes in the microbial communities associated with the floating plastics through metagenomic analysis (Yang et al., 2019), suggesting that the response to plastic pollution can be species-specific. Tetu et al. (2019), Sarker et al. (2020), and Piccardo et al. (2020) also revealed the importance of concentration levels of leached plastic in cyanobacteria and heterotrophic bacteria. Considering these previous results, experimental studies investigating the effect of MPs and their additives should take into account the response of different bacterial test species and different concentration levels of the pollutants. Moreover, the previous studies investigating the effect of plastic pollution use plastics with unknown chemical additives, and to separate the effects of plastics and additives, pure MPs and their known additives must be tested. Furthermore, varying physical properties of MPs have to be considered (e.g., density, hydrophobic, or size) since could affect the response of microorganisms.

Here, we report the responses of different bacterial species, specifically N_2_-fixing phototrophic and heterotrophic bacteria to different concentrations of pure MPs (i.e., PE, PP, PVC, and PS) and their most predominant organic chemical additives [fluoranthene, 1,2,5,6,9,10-hexabromocyclododecane (HBCD) and dioctyl-phthalate (DEHP)]. Our results show beneficial, detrimental or both effects, depending on the species tested and the type and concentrations of MPs and additives added.

## Materials and Methods

### Culture Strains Tested

Five marine N_2_-fixing species (two cyanobacteria and three heterotrophic bacteria), found in association with *P. oceanica* (Agawin et al., 2017; Fernández-Juárez et al., in prep), were selected according to the experimental design described in Supplementary Table 1. Before the experiments, the cells were acclimatized and cultured in their respective optimal culture media to achieve their exponential phase. Culture media were composed of synthetic seawater medium (ASN-III) + Turks island salts 4X for *Halothece* sp., BG11_0_ for *F. muscicola* and marine broth (MB) for the rest of the heterotrophic bacteria (Rippka et al., 1979). The cells were acclimated at 25°C at 120 r.p.m in a rotatory shaker with a photoperiod of 12 h (hours) dark:12 h light under low-intensity fluorescent light (30 μE m^−2^ s^−1^).

### Experimental Culture Conditions

All the experiments and response variables were performed in triplicate (*n* = 3) in artificial seawater (ASW) medium following Kim et al. (2007) at pH 7, adding 1 mL^−1^ per L of trace metal [L^−1^: 2.86 g H_3_BO_3_, 1.81 g MnCl_2_·4H_2_O, 0.22 g ZnSO_4_·7H_2_O, 0.39 g NaMoO_4_·2H_2_O, 0.079 g CuSO_4_·5H_2_O and 0.049 g Co(NO_3_)_2_·6H2O], glucose (final concentration 0.1% [v/v]), and with the respective MPs and/or organic additives. Inorganic phosphorus (${\text{PO}}_{4}^{3-}$, 0.04 g L^−1^ K_2_HPO_4_), iron (Fe, 0.006 g L^−1^ ferric citrate) and inorganic nitrogen (NH_3_, 0.5 g L^−1^ NH_3_Cl) were added according to the response variable measured as we described below. Bacteria at their exponential phase were inoculated in the treatments (Supplementary Table 1) and incubated for 72 h under the same conditions as when they were previously acclimatized (i.e., at 25°C, 120 r.p.m in a rotatory shaker with a photoperiod of 12 h dark:12 h light, under low-intensity fluorescent light, 30 μE m^−2^ s^−1^).

The pure MPs (low-density polyethylene [PE] with size 109 ± 6.29 μm, polypropylene [PP] with size 90 ± 7.56 μm and low-density polyvinyl chloride [PVC] with size 164 ± 8.03 μm) and organic additives (fluoranthene, 1,2,5,6,9,10-hexabromocyclododecane [HBCD] and dioctyl-phthalate [DEHP]) used were obtained from Sigma-Aldrich. Besides, we used fluorescent polystyrene (PS)-based latex beads (Fluoresbrite® YG Microspheres 1.00 μm, Polysciences, Inc.) as the lowest sized MPs, based on the definition of MPs (Hartmann et al., 2019). The stock solution of MPs was made at 100 mg mL^−1^, resuspending the MPs (previously UV-sterilized for 15 min) in acetone (98% [v/v]) to avoid aggregation of MPs and for easier manipulation of the workings solutions. To prevent chemical damages of MPs by acetone, the stock solution was rapidly diluted to working solutions of 1 mg mL^−1^ in ASW. The organic additives, i.e., fluoranthene and HBCD, were initially prepared in 1 mg mL^−1^ in absolute ethanol and acetone (98% [v/v]), respectively. For the DEHP additive that was in liquid form, a stock solution of 1 mg mL^−1^ was also prepared. We diluted these stock solutions to working solutions of 3 mg L^−1^ in ASW. Fluorescent PS beads were sterilized following the manufacturer's instructions, and the different concentrations in ASW were made from a stock solution of 4.55 × 10^10^ particles mL^−1^. The controls were made with the respective amounts of acetone and/or ethanol (without any MPs nor organic additives). All the treatments have ≤ 1% acetone or ethanol to avoid any biological effect in the cells, and co-solvents effect in water (Schwarzenbach et al., 2002).

Experiments were performed in two parts (Supplementary Table 1), i.e., (I) under environmentally relevant concentrations (in which we performed an initial screening of the five strains selected) and (II) the “worst-case” scenario (in which we selected two strains, one cyanobacterium and one heterotrophic bacterium as our model strains). Ecotoxicology thresholds for MPs were determined following (Reddy et al., 2006; Suaria et al., 2016; Everaert et al., 2018; Kane et al., 2020), which established that MPs can accumulate up to 29–133 μg mL^−1^ in the water column and seafloor. For organic additives, we followed the concentrations reported in Hermabessiere et al. (2017), in which it is reported that additives can accumulate between from pg L^−1^ to μg L^−1^, finding up to 44.39 μg L^−1^ in the water column.

#### Under Environmental Concentrations

In the first part, we made an overall screening of the five culture strains in sterile 2 mL well microplates (with 2 mL of culture media) to study their growth response under marine environmentally relevant concentrations of MPs and additives, with optimal nutrient conditions of ${\text{PO}}_{4}^{3-}$, Fe and NH_3_ (*n* = 3). We used five concentration for MPs (0, 0.01, 0.1, 1, and 100 μg mL^−1^) and organic additives (0, 0.3, 3, 30, and 300 μg L^−1^) (Supplementary Table 1). Additional treatments combining MPs and plastic additives (e.g., PE-fluoranthene, PP-HBCD, and PVC-DEHP) were done by combining the lowest and the highest number of MPs and additives to test for possible interacting effects of MPs and their organic additives (Supplementary Table 1). Growth analysis was assessed through flux cytometry (*n* = 3).

#### Under the “Worst-Case” Scenario

In the second part, we selected two strains, one autotrophic (*Halothece* sp., being our model phototrophic bacteria) and one heterotrophic (*Cobetia* sp., which was our model heterotrophic bacteria). We established two levels MPs and additives concentration: 100 and 1,000 μg mL^−1^ for MPs, and 300 and 3,000 μg L^−1^ for the organic additives. We also selected a concentration of 4.55 × 10^6^ and 4.55 × 10^7^ particles mL^−1^ for PS beads. We cultured the test bacteria in 50 mL falcon (with 30 mL of culture media) tubes under N_2_-fixing conditions (limiting NH_3_ [~ 0.15 mM] and with optimal ${\text{PO}}_{4}^{3-}$ and Fe) for growth, protein overproduction, microscopical analysis, ${\text{PO}}_{4}^{3-}$-uptake and N_2_-fixation assays, or under ${\text{PO}}_{4}^{3-}$-limiting conditions (with optimal NH_3_ and Fe) for alkaline phosphatase activity (APA) (*n* = 3, Supplementary Table 1).

### Flow Cytometry

Aliquots of cultures from all the experiments were taken initially and after 72 h of incubation and counted in fresh (without freezing nor fixing) with a Becton Dickinson FACS-Verse cytometer (Beckton & Dickinson, Franklin Lakes, New Jersey, USA). Fluorescent beads, BD FACSuite™ CS&T research beads (Beckton & Dickinson and Company BD Biosciences, San Jose, USA), were used as internal standards to calibrate the instrument. Cells were separated by combinations of the scatter plots of the flow cytometer parameters: forward scatter (FSC, reflecting cell size), side scatter (SSC, reflecting internal complexity of the cells), and/or fluorescein isothiocyanate filter (FITC, reflecting fluorescence, 488 nm excitation, 530/30 nm emission). For treatments with fluorescent PS beads, adsorption to them was measured (without detaching cells from the beads) using the FSC and FITC cytometer signals (Supplementary Figure 1). Adsorbed bacteria were those with intermediate intensity fluorescence signals between the free bacteria and the beads (Supplementary Figure 1). In all the experiments, a total of 10,000 cells (or cells recorded in 30 s) were counted in each sample. Changes in growth were calculated as the changes in cell concentrations after 72 h.

### Protein Identification: MALDI-TOF Assay and Protein Structure Prediction

Crude cell extracts were done following the methods described in Ivleva and Golden (2007), using the cultures of *Halothece* sp. and *Cobetia* sp. after 72 h of incubation in the “worst-case” scenario (at the highest concentration treatment of MPs/additives and the control). Protein concentrations were determined with Bradford protein assay (Thermofisher), following the manufacturer's instructions. The protein extracts were run into polyacrylamide gels, 4–20% (p/v) Amersham ECL Gel (GE Healthcare, Chicago, Illinois, EEUU), using the ECL Gel Box system (GE Healthcare, Chicago, Illinois, EEUU) following the manufacturer's instructions.

The different bands detected only in *Cobetia* sp. exposed to high concentrations of MPs (1,000 ug mL^−1^ of PE, PP, and PVC), which did not appear at the control, were excised from the gel with a clean scalpel and sent to MALDI-TOF analysis. Each gel slice was cut into small pieces and then transferred to a clean and sterile Eppendorf tube. Protein identification was performed following Jaén-Luchoro et al. (2017). The samples were then analyzed with an Autoflex III MALDI-TOF-TOF (BrukerDaltonics) spectrometer using the software Compassflex series v1.4 (flexControl v3.4, flexAnalysis v3.4 and BioTools 3.2). The spectra were calibrated using the peptide calibration standard (BrukerDaltonics). The obtained mass spectra were used for the protein identification and the in-house database was created with the predicted protein sequence of the annotated genome of *Cobetia* sp. The search process was performed with the algorithm MASCOT (MatrixSciences).

Fasta sequence of the alcohol dehydrogenase (ADH) (detected through MALDI-TOF) was sent to the I-Tasser server for protein 3D-structure prediction (Zhang, 2008), with their domains previously checked in Pfam 32.0 (Finn et al., 2016). The predicted structure was sent to the FunFOLD2 server for the prediction of protein–ligand interactions (Roche et al., 2013). Besides, we detected the potential sites of ligand or “pockets” through MetaPocket 2.0 (Huang, 2009). Finally, we predicted the orientation and position of the protein-ligand complex between polyethylene glycol (PEG) and ADH, docking these with Swissdock (Zoete and Michielin, 2011). All the structures were visualized by Pymol (DeLano, 2002).

### Microscopic Observations

At the final time (after 72 h), the five strains tested were placed onto a microplate for inverted microscopy visualization of the physical interactions between bacterial cells and the MPs (i.e., PE, PP, and PVC) at 100x objective (Leica DM IRB). For *Halothece* sp. and *Cobetia* sp., their interaction with PS fluorescent beads (with an excitation of 441 nm and emission of 485 nm) were visualized by confocal microscopy (Leica TCS SPE, Leica Microsystems). Images were processed using the software Leica application suite (Leica Microsystems). The specific autofluorescence for *Halothece* sp. was observed with an excitation of 532 nm and an emission of 555–619 nm. For *Cobetia* sp., the cells were stained with Sybr green (Sigma-Aldrich), or propidium iodide (Sigma) to properly visualize the cells distinguishing them from the PS beads.

### P-Metabolism Analysis

Alkaline phosphatase activity (APA) was evaluated through fluorometric assay following the hydrolysis of the substrate (S) 4-methylumbelliferyl phosphate (MUF-P, Sigma-Aldrich) to 4-methylumbelliferyl (MUF), following Fernández-Juárez et al. (2019). The culture media, i.e., in the “worst-case” scenario, was ${\text{PO}}_{4}^{3-}$ limited (but with optimal NH_3_ and Fe) to promote APA. Saturation curves of velocity (V, fmol MUF cell^−1^ h^−1^) vs. substrate (S, μM) were made for each experimental condition for each of the two selected strains (*Halothece* sp. and *Cobetia* sp.). We used different concentrations of MUF-P: 0, 0.05, and 5 μM of MUF-P. After 1 h incubation in darkness at room temperature, APA was measured in a microtiter plate that contained buffer borated pH 10 (3:1 of sample: buffer). The MUF production (fmol MUF cell^−1^ h^−1^) was measured with a Cary Eclipse spectrofluorometer (FL0902M009, Agilent Technologies) at 359 nm (excitation) and 449 nm (emission), using a calibration standard curve with commercial MUF (Sigma-Aldrich). The maximum velocity (V_max_) at saturating substrate concentrations was obtained from each plot of V vs. S, using the Lineweaver-Burk plot.

For the determination of inorganic ${\text{PO}}_{4}^{3-}$ concentrations for *Halothece* sp. and *Cobetia* sp. experiments, 1 mL of the culture was centrifuged for 15 min at 16,000 × g under 4°C. The bacteria-free clear supernatant was collected and used for ${\text{PO}}_{4}^{3-}$ determinations following standard spectrophotometric methods (Hansen and Koroleff, 2007). The ${\text{PO}}_{4}^{3-}$ concentrations in the culture media were determined at the initial and final time (after 72 h). Specific ${\text{PO}}_{4}^{3-}$-uptake rates (pmol ${\text{PO}}_{4}^{3-}$ cell^−1^ d^−1^) were calculated as described in Fernández-Juárez et al. (2019) and Ghaffar et al. (2017).

### Determination of N_2_-Fixation Rates Through Acetylene Reduction Assay (ARA)

Rates of N_2_-fixation were measured for *Halothece* sp. as our model strain, described in Fernández-Juárez et al. (2019, 2020), under the “worst-case” scenario. A known volume of culture (8 mL) was sampled during the dark photoperiod and transferred to a closed hermetic vial and oxygen was flushed from the sample through bubbling with N_2_ gas. A medium with saturated acetylene was injected at 20% (v/v) final concentration in each vial with a sterile syringe. Samples were incubated for 3 h at room temperature (24°C) in the dark. After the 3 h incubation time, 10 mL of liquid was removed and transferred to Hungate tubes containing 1.25 mL of 20% trichloroacetic acid (Agawin et al., 2014). Prior to analysis with the gas chromatograph (GC), the Hungate tubes were incubated at 37°C overnight in a water bath. Ethylene and acetylene gas from the gas phase of the Hungate tubes were determined using a GC (model GC-7890, Agilent Technologies) equipped with a flame ionization detector (FID), following the set up described in Fernández-Juárez et al. (2019, 2020). Ethylene produced was calculated using the equation in Stal (1988). The acetylene reduction rates were converted to N_2_-fixation rates (nmol mL^−1^ h^−1^) using a factor of 4:1 (Jensen and Cox, 1983).

### Statistical Analysis

Non-normally distributed data, the Kruskal-Wallis rank-sum non-parametric test was used since the size sample was *n* < 20. An unpaired two-sample Wilcoxon test was used to determine the significant effects among different concentrations of MPs and additives. All analyses were done in R-Studio, R version 3.5.3 (2019-03-11).

## Results and Discussion

### Effects of Varying Concentrations of MPs and Additives, Under Relevant Concentrations in the Marine Environment

**Figure 1:**
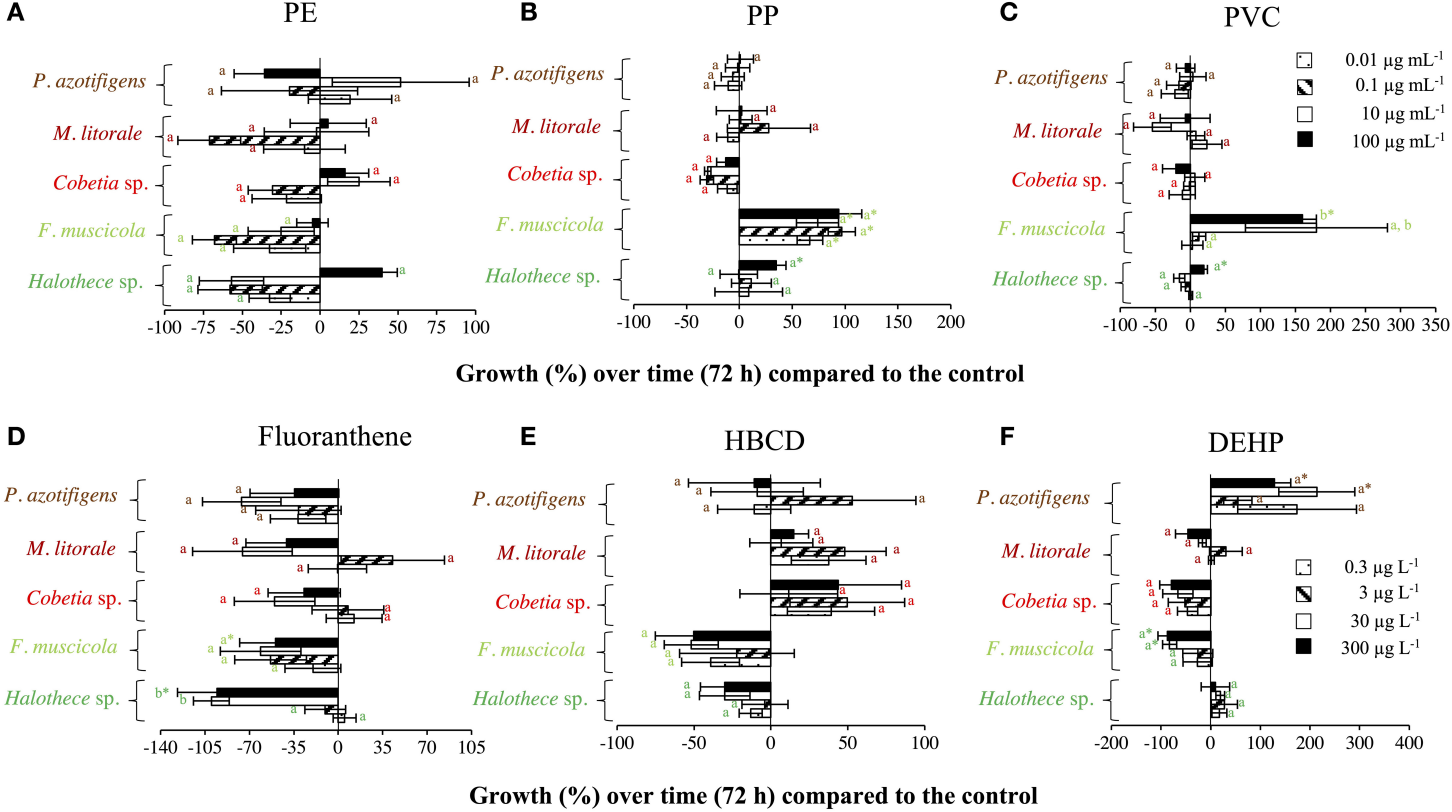
Growth responses of diazotrophs subject to MPs and organic additives under relevant concentrations in the marine environment after 72 h, compared with the control value. **(A)** PE, **(B)** PP, and **(C)** PVC, with 0, 0.01, 0.1, 10, and 100 μg mL^−1^. **(D)** Fluoranthene, **(E)** HBCD, and **(F)** DEHP, with 0, 0.3, 3, 30, and 300 μg L^−1^. Values are the mean, and the error bar is the standard error between the replicates (*n* = 3). Letters indicate pairwise analysis among the variables (i.e., concentration) inside each strain (see the different colors), and asterisks (*) indicate pairwise significant differences between variables and the control (*p* < 0.05), using a *post-hoc* test (Wilcoxon) after Kruskal-Wallis over the whole dataset.

The addition of PE within the range of 0–100 μg mL^−1^ did not significantly affect all the diazotrophs tested (*p* > 0.05, *n* = 3, Figure 1A), in agreement with Machado et al. (2020). Moreover, PP and PVC within the range of 0–100 μg mL^−1^ did not significantly affect the growth of heterotrophic bacteria (*Cobetia* sp., *Marinobacterium litorale* and *Pseudomonas azotifigens*) (*p* > 0.05, *n* = 3, Figures 1B,C). This is consistent with the results obtained by Piccardo et al. (2020), in which PET microparticles at 100 μg mL^−1^ do not have affect in the heterotrophic bacteria (e.g., *Vibrio fischeri*). However, species-specific growth responses of the bacteria tested with the addition of MPs were exemplified here when PVC and PP addition (at 100 μg mL^−1^) significantly enhanced the growth of the autotrophic cyanobacterial diazotrophs (*Halothece* sp. and *Fischerella muscicola*) by 1.5- to 4- fold (*p* < 0.05, *n* = 3, Figures 1B,C). This supports the idea that MPs may be selecting bacterial communities in the ocean (Seeley et al., 2020).

**Figure 2:**
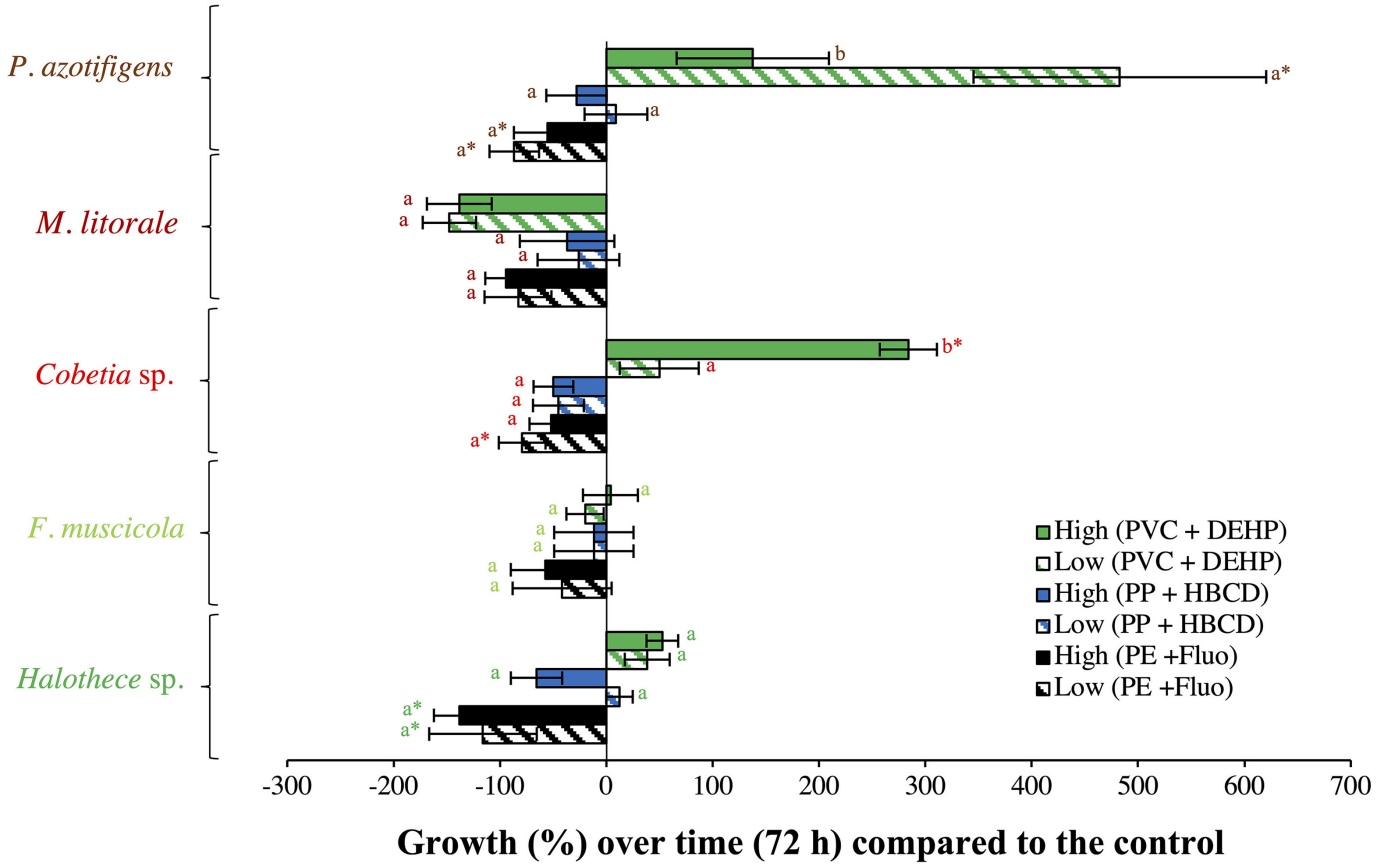
Growth responses of diazotrophs subject to MP-additive interactions (PE + fluoranthene, PP **+** HBCD, and PVC + DEHP) after 72 h (under relevant concentrations), compared with the control value. “Low” and “High” represent low and high concentrations of MPs (0.01 and 100 μg mL^−1^, respectively) and plastic additive (0.3 and 300 μg L^−1^, respectively). Values are the mean, and the error bar is the standard error between the replicates (*n* = 3). Letters indicate pairwise analysis among the variables (i.e., concentration) inside each strain (see the different colors), and asterisks (*) indicate pairwise significant differences between variables and the control (*p* < 0.05), using a *post-hoc* test (Wilcoxon) after Kruskal-Wallis over the whole dataset.

The effect of organic additives on bacterial growth is suggested here to be dependent on the type of additive (i.e., fluoranthene, HBCD and DEHP) (Figures 1D–F). Fluoranthene reduced the growth of *Halothece* sp. and *F. muscicola*, by 22- and 7- fold, respectively, at the highest concentrations compared to the control (*p* < 0.05, *n* = 3, Figure 1D). Besides, DEHP significantly reduced the growth of *F. muscicola* at the highest concentrations compared to the control (*p* < 0.05, *n* = 3, Figure 1F). On the contrary, the additive HBCD did not affect the growth of any species (*p* > 0.05, *n* = 3, Figure 1E), while the additive DEHP significantly enhanced the growth of the heterotrophic *P. azotifigens* by 4-fold, being significative at 30 and 300 μg L^−1^ compared to the control (*p* < 0.05, *n* = 3, Figure 1F). Additives can be sorbed and/or liberated by MPs in marine environments, with contrasting consequences (Gallo et al., 2018; Hahladakis et al., 2018). If sorbed, these chemicals may be less available for cells, being less harmful to sensitive species (Hahladakis et al., 2018), but detrimental to species making use of them as a C-source (Cao et al., 2015; Wang et al., 2015). If liberated, the increased availability of additives may be more harmful to sensitive species (Tetu et al., 2019; Sarker et al., 2020), but benefit species using them (Cao et al., 2015; Wang et al., 2015). In our experiments, PVC + DEHP significantly enhanced the growth of *P. azotifigens* and *Cobetia* sp. (synergism), but significantly altered it when PE + fluoranthene were added (antagonism) (*p* < 0.05, *n* = 3, Figure 2), while the addition of PP + HBCD did not have any further effect (*p* > 0.05, *n* = 3, Figure 2).

### Effect of Varying Concentrations of MPs and Additives, Under the “Worst-Case” Scenario

#### Effect of High Concentration of MPs

**Figure 3:**
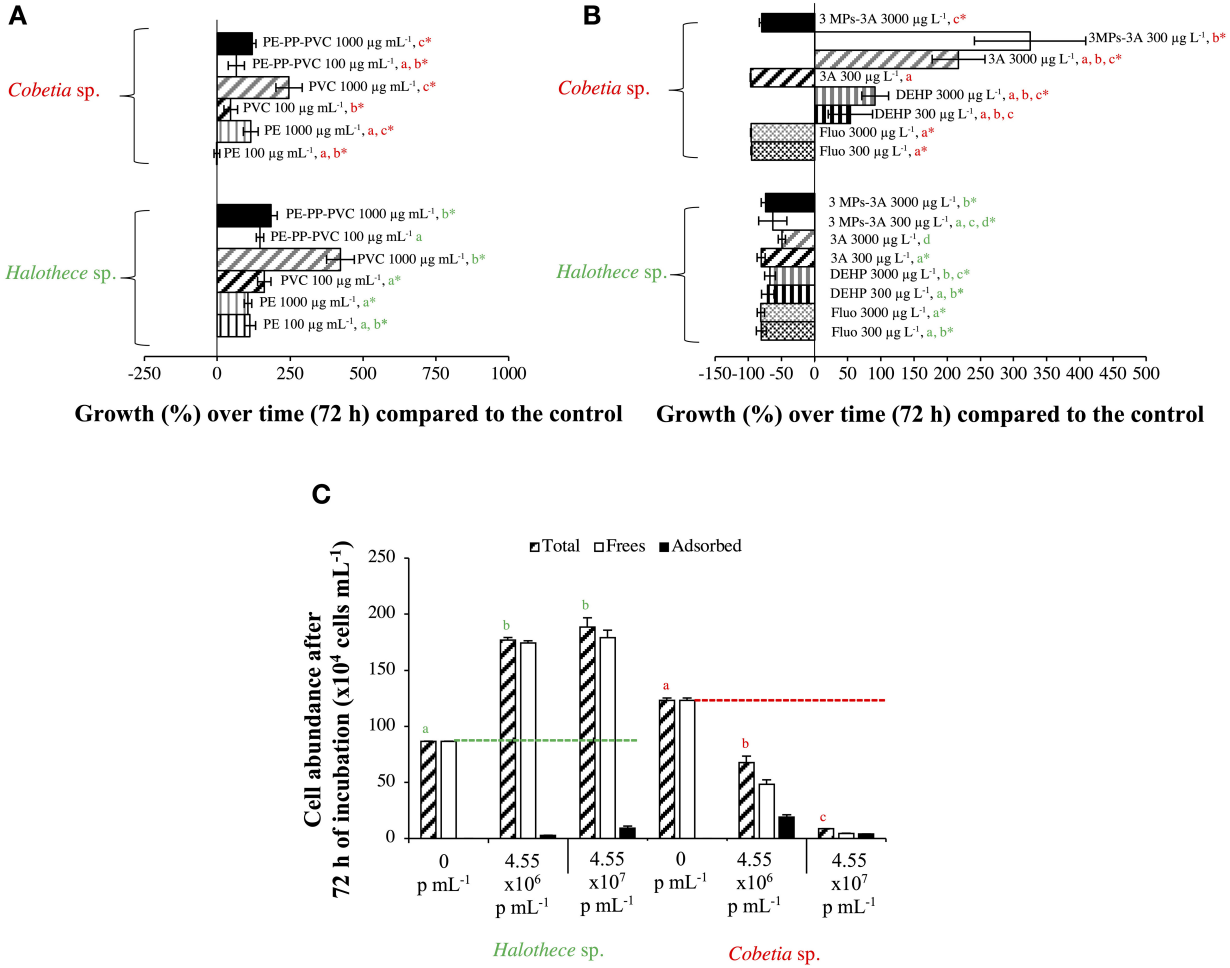
Growth responses of diazotrophs subject to high concentrations (the “worst-case” scenario) of MPs and additives after 72 h, compared with the control value. **(A)** Effect of MPs (PE, PP, and/or PVC, 100/1,000 μg mL^−1^). The effect of PP alone with non-significant results is not included in the graph. **(B)** Effect of organic additives (fluoranthene, HBCD, and/or DEHP, 300/3,000 μg L^−1^). The effect of HBCD alone with non-significant results is not included in the graph. **(C)** Effect of PS beads. Total, free and adsorbed bacteria (to the beads) are differentiated. Values are the mean, and the error bar is the standard error between the replicates (*n* = 3). Letters indicate pairwise analysis among the variables (i.e., concentration) inside each strain (see the different colors), and asterisks (*) indicate pairwise significant differences between variables and the control (*p* < 0.05), using a *post-hoc* test (Wilcoxon) after Kruskal-Wallis over the whole dataset.

High MPs concentrations stimulated cell growth, especially with the addition of PVC in both strains compared with the control by 6- to 8- fold (*p* < 0.05, *n* = 3, Figure 3A). The enhancement of the bacterial growth, especially in the heterotrophic strain, *Cobetia* sp., after 72 h of incubation can be due to increased dissolved organic carbon (DOC) pool in the medium that could have leached from the MPs added (Romera-Castillo et al., 2018). Unfortunately, DOC was not measured.

**Figure 4:**
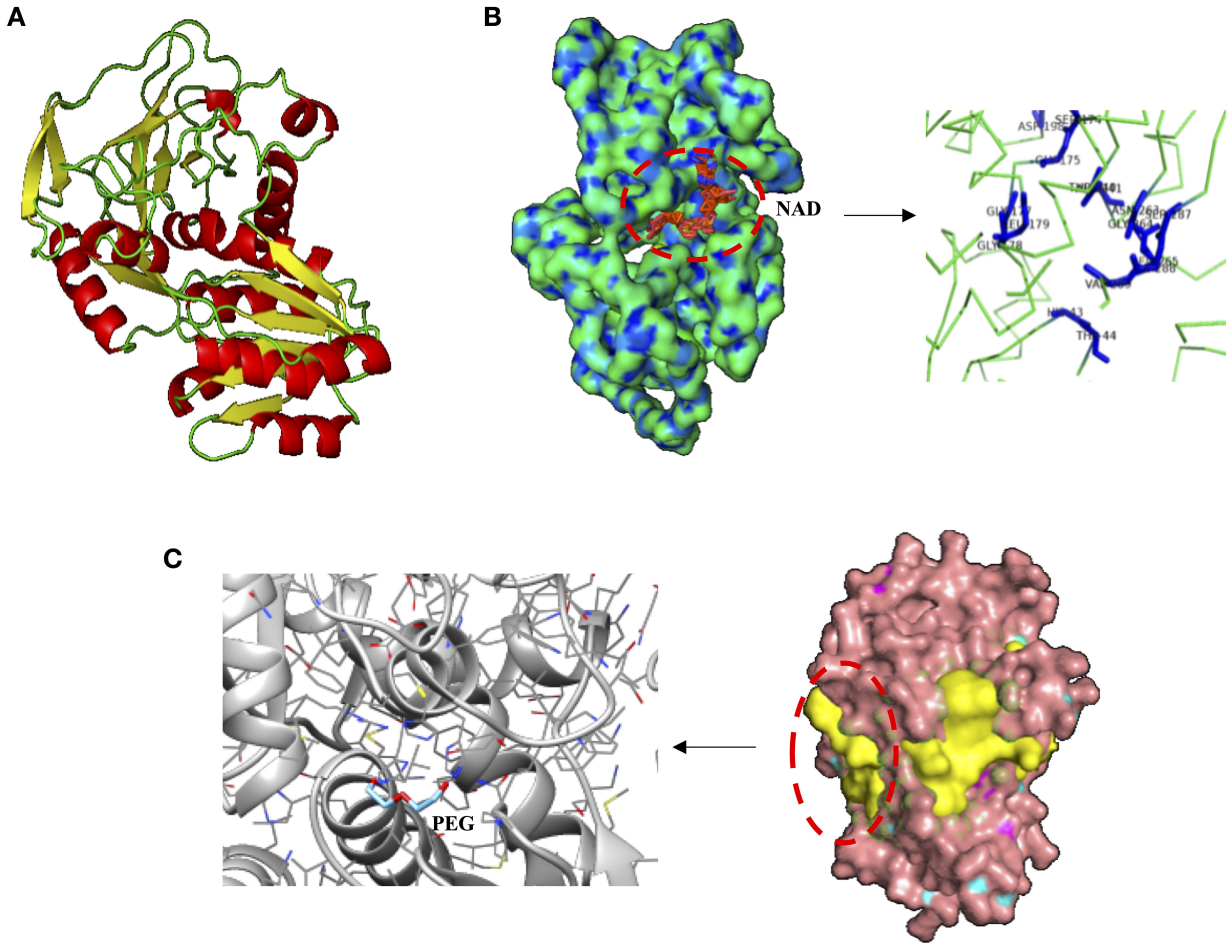
Structural analysis of alcohol dehydrogenase (ADH) from *Cobetia* sp. (HA399_02440). **(A)** Protein prediction of ADH showing, **(B)** the nicotinamide-adenine-dinucleotide (NAD) dependence, displaying the ligand binding residues HIS 43, THR 44, SER 174, GLY 175, GLY 177, GLY 178, LEU 179, ASP 198, THR 240, ASP 241, ASN 263, GLY 264, LYS 265, SER 287, ILE 288, and VAL 289. **(C)** The three of the most probable pockets of the ADH (potential sites of ligand binding, in yellow), and the docking site (prediction of the orientation and position of protein-ligand complex) between the polyethylene glycol (PEG) and ADH.

Species-specific responses based on protein production profile are also shown here when two proteins related to plastic degradation (alcohol dehydrogenase [ADH] of 342 amino acids, HA399_02440) and carbon transport (C4-dicarboxylate ABC transporter substrate-binding protein [C4-ABCS] of 329 amino acids, HA399_06715) were overproduced in the heterotroph *Cobetia* sp., but were not detectable with the MALDI-TOF analyses in the cyanobacteria *Halothece* sp. after the addition of MPs at high concentrations. ADHs from *Rhodopseudomonas acidophila* M402 and *Pseudomonas oleovorans* have been shown to oxidize plastic polymers (e.g., PEG), being NAD-dependent (Ohta et al., 2006; Kawai, 2010), consistent with our *in silico* structural analysis for *Cobetia* sp. (Figures 4A–C). Further experimental studies have to be performed to evaluate if *Cobetia* sp. can degrade PEG polymers or similar ones, and indeed if the carbon released by ADH is transported by C4-ABCS inside the cells since C4-ABCS is a carbon transporter (Rosa et al., 2019).

##### Effect of the Size of MPs

**Figure 5:**
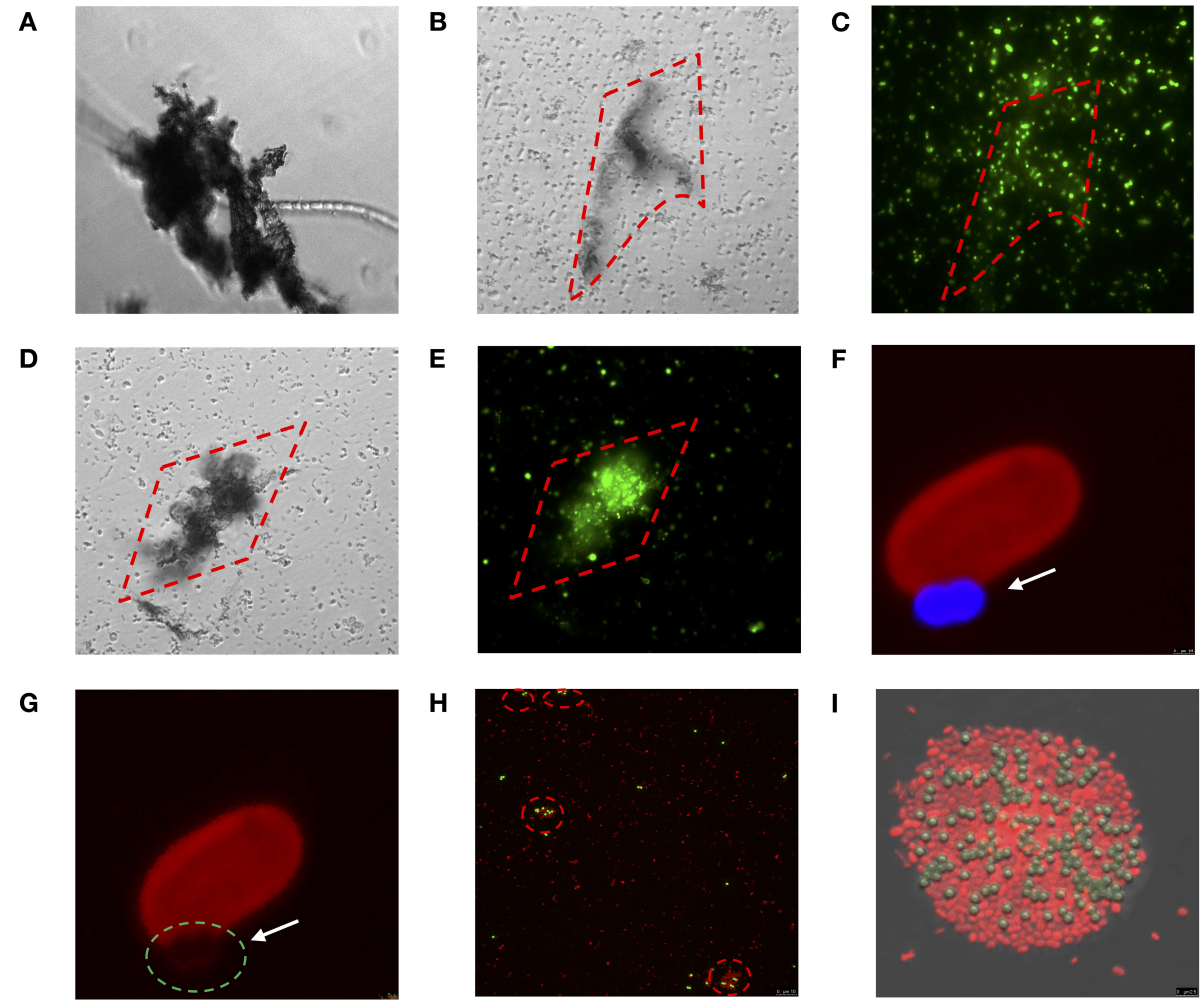
Direct physical interaction between MPs and bacterial species tested in which **(A–E)** show the interaction between diazotrophs and mix of MPs (PE, PP, and PVC) at 100 μg mL^−1^ through inverted microscopy: **(A)** *Fischerella muscicola* under bright-field (BF), **(B,C)** *Cobetia* sp. under BF and Sybr-green channel, respectively, **(D,E)** *Marinobacterium litorale* under BF and Sybr-green channel, respectively; **(F–I)** show the physical interaction between diazotrophs and PS 1 μm beads (excitation of 441 nm and emission of 485 nm) through confocal microscopy: **(F,G)** *Halothece* sp. under excitation of 532 nm wavelength and emission of 555–619 nm. The arrows and green dashed circle show where the cell membrane is interacting with the PS bead (blue). **(H,I)** *Cobetia* sp. under excitation of 493 and 636 nm of emission. The dashed red circles show cell agglomeration on the PS beads (yellow). Images were taken at 1000x **(A**–**E** and **H)** and further magnified **(F,G,I)**.

Larger-sized MPs (i.e., 120 μm) enhanced the growth of autotropic and heterotrophic bacteria at high concentrations of MPs (Figure 3A). Larger-sized MPs may provide more surface area for the cells to adhere and attach as observed in the heterotrophs tested here (Figures 5B–E). Surface attachment of the cells to the MPs through hydrodynamic and electrostatic interactions may enhance growth and facilitate nutrient uptake (especially under oligotrophic conditions), in most of the cases by the biofilm formation, increasing the surface of the substrates. This can aid the uptake of the necessary metabolites and co-factors, as suggested in Tuson and Weibel (2013). Smaller-sized MPs (i.e., PS beads of 1 μm-size), however, affected negatively the smallest size heterotrophic bacteria (i.e., *Cobetia* sp. of ~ 1 μm-size, *p* < 0.05, *n* = 3, Figure 3C), and not the unicellular cyanobacteria (i.e., *Halothece* sp. of ~ 4 to 7 μm-size) (*p* > 0.05, *n* = 3, Figure 3C). This can be due to the differences in the degree of physical adsorption between the PS beads and the different bacterial species. Approximately 40–87% of *Cobetia* sp. cells were adsorbed to PS (Figures 3C, 5H,I), while only ~2–5% of *Halothece* sp. cells were adsorbed (Figure 3C). The mechanisms behind the negative effect of small-sized MP in small-sized bacteria needs to be further studied and can possibly be due to disruption of bacterial cell division by the aggregation of the cells and beads. Although fewer PS beads were adsorbed on *Halothece* sp. cells, an invagination of the cell membranes by PS beads has been observed, possibly being engulfed or included as a carbon source (Figures 5F,G).

##### Physicochemical Properties of MPs

The physicochemical properties (e.g., hydrophobicity, electrostatic attraction, or roughness) of different MPs may affect the responses of bacterial communities (Ogonowski et al., 2018). Hydrophobicity, for example, can directly affect the bacterial colonization of MPs. PS polymers, which have aromatic phenyl groups, are one of the most hydrophobic polymers (Ogonowski et al., 2018), and this may explain the adherence of both autotrophic and heterotrophic bacteria to PS beads (Figures 5F–I). Contrary to the cyanobacterial diazotrophs (*Halothece* sp. and *F. muscicola*), which were not capable of adhering to the MPs, heterotrophic N_2_-fixers (*Cobetia* sp., *M. litorale*, and *P. azotifigens*) tested were able to adhere to other types of MPs (i.e., PE, PP, and PVC, Figures 5A–E) which are less hydrophobic than PS. Moreover, PVC polymers are slightly more hydrophilic than PE (Kennedy, 2014), suggesting that they can be less available for adhesion and more available for bacterial growth. This may explain why PVC polymers were the MPs that most enhanced bacteria growth (Figure 3A).

#### Effect of High Concentrations of Organic Additives

Contrary to the effects of MPs, the addition of different types of organic additives (fluoranthene, HBCD and/or DEHP up to 3,000 μg L^−1^) affected negatively the growth of *Halothece* sp. by 8-fold (*p* < 0.05, *n* = 3, Figure 3B). For the heterotrophic bacteria, different responses were observed, being the fluoranthene the most toxic pollutant (*p* < 0.05, *n* = 3, Figure 3B). The differing sensitivities of different species of bacteria to a particular type of additive can be due to cell-size dependent toxicity. For example, here we found an increasing toxicity to a PAH additive, fluoranthene, from bigger- to smaller-sized: *Cobetia* sp. (~1 μm) > unicellular cyanobacteria *Halothece* sp. (4–7 μm), > filamentous cyanobacteria *F. muscicola* (~7 μm) (*p* < 0.05, Spearman's correlation, *n* = 21, *r*^2^ = 0.7) (Figures 1D, 3B). The negative correlation between cell size and PAH toxicity is consistent with the study of Echeveste et al. (2010), and may be due to the higher surface to volume ratio of smaller-sized cells which increases the potential of the additives to be adsorbed or consumed by the cells. The DEHP additive (at high concentrations) and the interaction of the three MPs with the three plastic additives at low concentrations enhanced the growth of *Cobetia* sp. (*p* < 0.05, *n* = 3, Figure 3B), indicating that this species might use DEHP as a C-source and can be a possible bioremediator of DEHP-contaminated environments like *Rhodococcus* (Wang et al., 2015). Nevertheless, here we did not intend to reproduce an environmental situation since it may be improbable to find such high concentrations in the water column due to their solubility. However in marine sediments, concentrations of up to 2,988 μg Kg^−1^ of organic pollutants can be found in extremely impacted areas (Hermabessiere et al., 2017), and thus our results provide useful data to understand the response of the microorganisms associated with the benthic organisms.

### Effect of MPs Pollution in the P and N-Metabolism

#### P-Acquisitions Mechanisms

**Figure 6:**
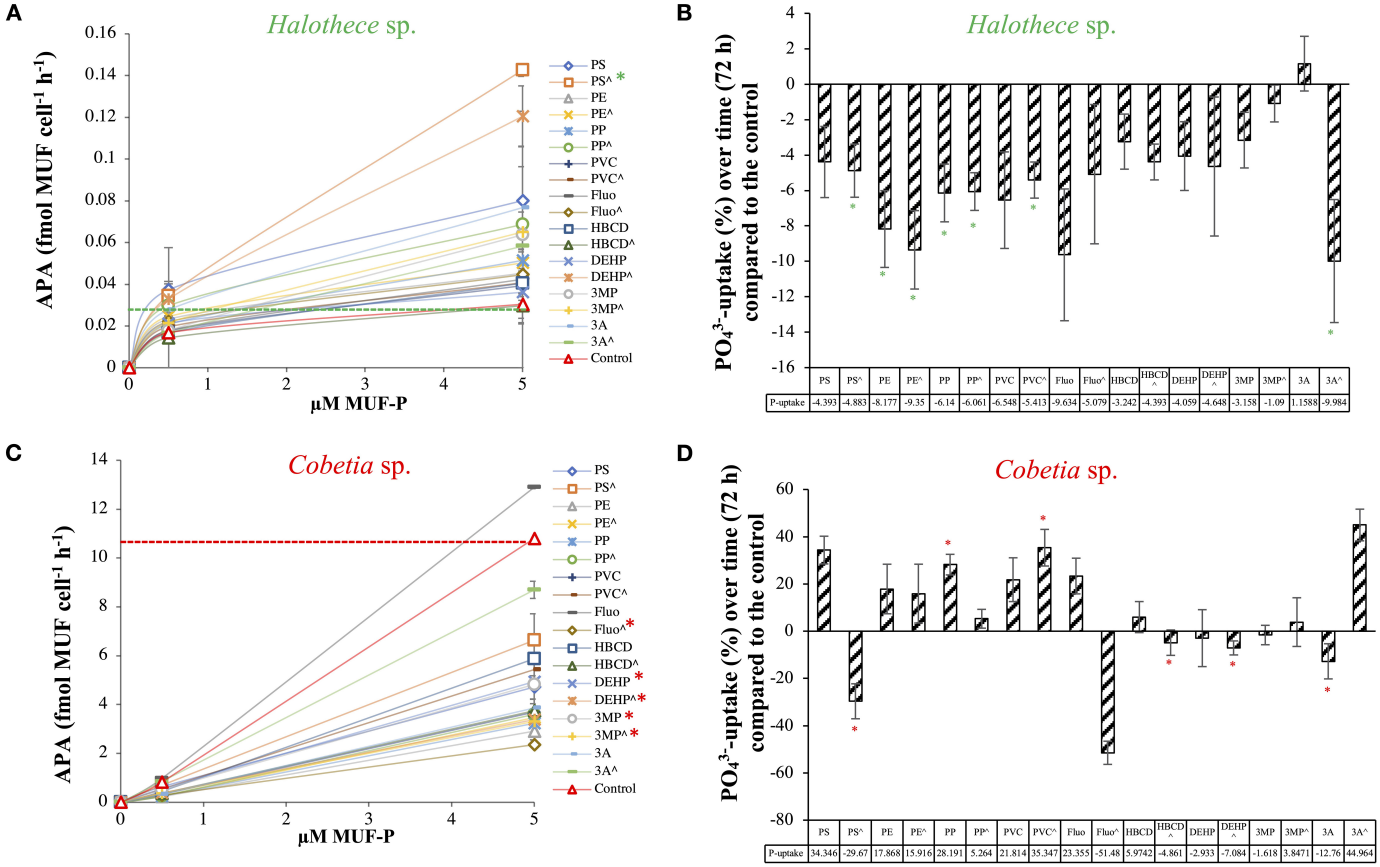
Phosphorus acquisition mechanisms for two species of N_2_-fixers exposed to different MPs and their plastic additives: **(A)** Alkaline phosphatase activity (APA, fmol MUF cell^−1^ h^−1^) and (**B)** ${\text{PO}}_{4}^{3-}$-uptake (pmol ${\text{PO}}_{4}^{3-}$ cell^−1^ d^−1^) for *Halothece* sp., using relative values (with the control as reference). **(C)** APA and **(D)** ${\text{PO}}_{4}^{3-}$-uptake for *Cobetia* sp., using relative values (with the control as reference). (∧) indicates treatments up to 1,000 μg mL^−1^ (MPs) and 3,000 μg L^−1^ (organic additives), without (∧) indicates treatments with 100 μg mL^−1^ (MPs) and 300 μg L^−1^ (organic additives). Values are the mean, and the error bar is the standard error between the replicates (*n* = 3). Asterisks (*) indicate significant differences (*p* < 0.05) compared with the controls, using a *post-hoc* test (Wilcoxon) after Kruskal-Wallis over the whole dataset.

The MPs and their plastic additives generally enhanced the alkaline phosphatase activity (APA) of *Halothece* sp. The addition of PS beads (at 4.55 × 10^7^ particles mL^−1^) increased the maximum rate of reaction (V_max_) up to 0.21 fmol MUF cell^−1^ h^−1^, significantly higher than controls with a V_max_ of 0.033 fmol MUF cell^−1^ h^−1^ (*p* < 0.05, *n* = 3, Figure 6A). In previous experiments, we described that the cyanobacterial N_2_-fixer *Halothece* sp. synthesizes an alkaline phosphatase D (PhoD) that is Fe dependent (Fe as metal co-factor) (Fernández-Juárez et al., 2019). Since metals (i.e., Fe) can be accumulated onto the plastics (Rochman et al., 2014), it is hypothesized that MPs may promote an environment rich in Fe co-factors. Considering the P-dependence of important processes (e.g., N_2_-fixation, Fernández-Juárez et al., 2019), stimulation of APA can promote the growth of *Halothece* sp. For this unicellular cyanobacterium, ${\text{PO}}_{4}^{3-}$-uptake rates were significantly downregulated by the addition of MPs and their plastic additives (*p* < 0.05, *n* = 3, Figure 6B). Comparisons between treatments were made (Supplementary Table 2), showing that the combination of the three MPs (i.e., high levels) and the three additives (i.e., low levels) were the treatments with lower reduction of the ${\text{PO}}_{4}^{3-}$-uptake. The decreased ${\text{PO}}_{4}^{3-}$-uptake rates observed in *Halothece* sp. may be due to the adsorption of phosphate ions by PE and PVC (Hassenteufel et al., 1963).

Unlike *Halothece* sp., APA for *Cobetia* sp. was generally reduced by MPs and their organic additives (*p* < 0.05, *n* = 3, Figure 6C). Among the MPs, PE addition at high concentrations caused the most significant decrease in APA (V_max_ = 8.52 fmol MUF cell^−1^ h^−1^) compared to controls (V_max_ = 32.78 fmol MUF cell^−1^ h^−1^) (*p* < 0.05, Figure 6C). Among the plastic additives, fluoranthene caused the highest decrease in APA (V_max_ = 11.52 fmol MUF cell^−1^ h^−1^) compared to controls (*p* < 0.05, Figure 6C). Significant differences in ${\text{PO}}_{4}^{3-}$-uptake rates were observed among the treatments tested (*p* < 0.05, *n* = 3, Figure 6D and Supplementary Table 2). Contrary to *Halothece* sp., ${\text{PO}}_{4}^{3-}$-uptake was increased with the addition of PP and PVC (*p* < 0.05, *n* = 3, Figure 6D), maybe as a consequence of higher nutrient and energy requirements for growth. Hence, we show species-specific differences of the P-mechanisms and P-requirements and the responses of these processes to MPs and their additives, showing that P-homeostasis can be disturbed with the addition of MPs and their organic additives associated.

#### Effect on N_2_-Fixation in Cyanobacteria

**Figure 7:**
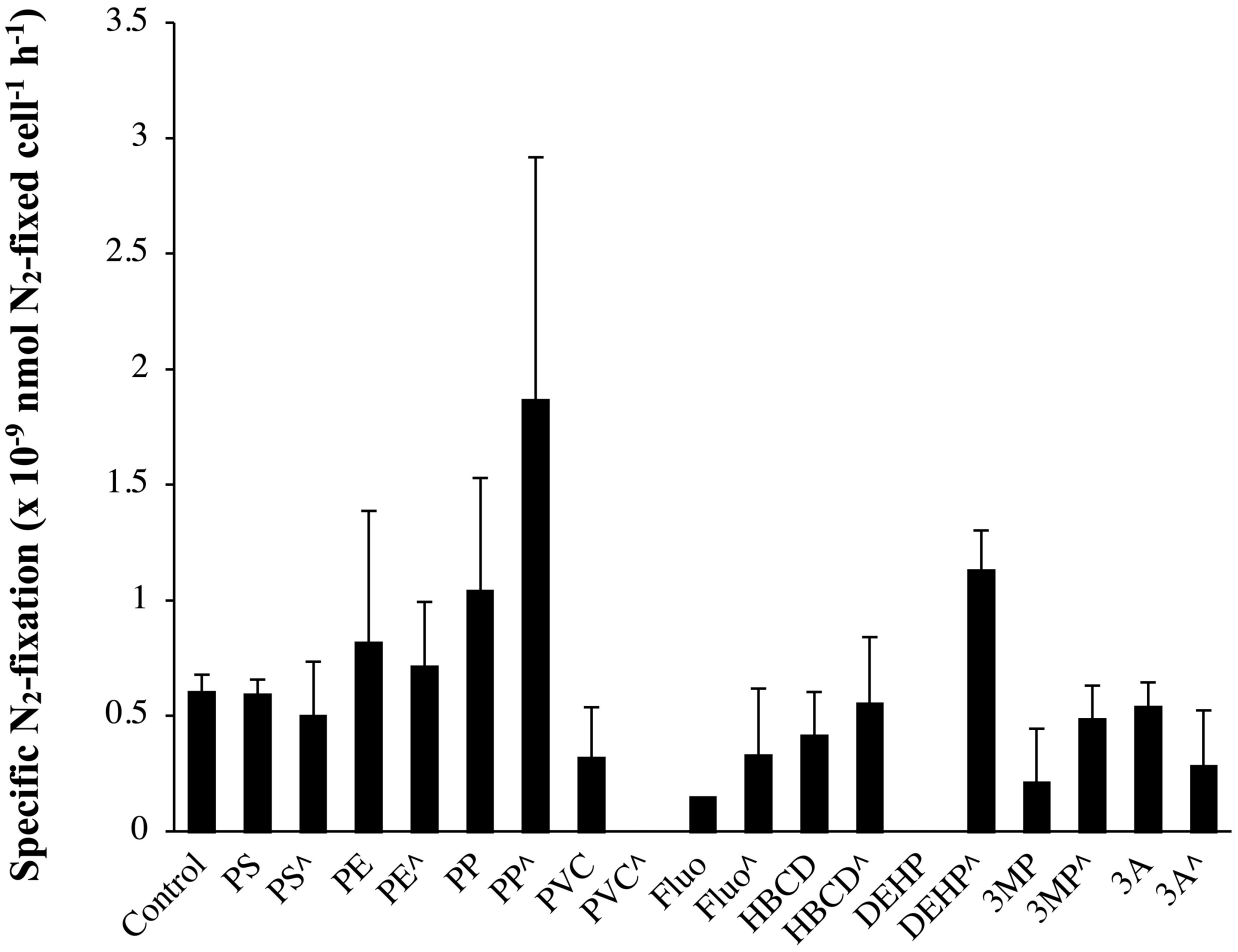
Specific N_2_-fixation rates (nmol N_2_-fixed cell^−1^ h^−1^) in the unicellular cyanobacteria *Halothece* sp. exposed to MPs and their plastic additives. (∧) indicates treatments up to 1,000 μg mL^−1^ (MPs) and 3,000 μg L^−1^ (organic additives), and without (∧) indicates treatments with 100 μg mL^−1^ (MPs) and 300 μg L^−1^ (organic additives). Values are the mean, and the error bar is the standard error between the replicates (*n* = 3).

In a seminal paper, Bryant et al. (2016) claimed that MPs may be hot spots of N_2_-fixing autotrophic bacteria, based on the high abundances of N_2_-fixation genes (*nifH, nifD*, and *nifK*) in the metagenomes associated with the plastic. Unfortunately, the authors did not measure the N_2_-fixation activities, considering that N_2_-fixation rates in the open ocean are largely maintained by cyanobacteria (Zehr and Capone, 2020). Hence, cyanobacteria N_2_-fixers can be one of the most impacted groups. Here, the effects of MPs and their additives on N_2_-fixation rates are reported for the first time in cyanobacteria (i.e., *Halothece* sp., Figure 7). However, MPs and their additives did not have a significant effect on specific N_2_-fixation rates of *Halothece* sp. (*p* > 0.05, *n* = 3, Figure 7), but as we showed that growth was positively enhanced with the addition of MPs (Figure 3A), or negatively affected by the addition of organic additives (Figure 3B), these pollutants could eventually enhance/inhibit global N_2_-fixation rates in the environment.

In summary, this study shows that the most predominant MPs (e.g., PE, PP, PVC, and PS) in the oceans and their commonly associated organic additives (i.e., fluoranthene, HBCD, and DEHP) can be beneficial (the “good”), deleterious (the “bad”), or both (the “double-sword”) to marine bacteria. Our study provides useful data to understand the response of marine bacteria, especially the diazotrophs to MPs pollution. Nevertheless, the transposition of the results obtained under *in vitro* controlled conditions must be taken with precautions since our study used concentrations that may not be representative of all marine environments. Open questions such as how the hydrophobicity of MPs can affect the growth responses, or if N_2_-fixers may have another important environmental role of biodegrading synthetic plastic polymers aside from their important ecological role of providing new N into marine ecosystems, have to be addressed. The use of next-generation analysis (i.e., transcriptomic or proteomic assays) to identify changes in gene expression or protein profiles derived from MPs and plastic additives may allow a better comprehension of the molecular responses behind the plastic threat in oceans.

## Data Availability Statement

The datasets presented in this study can be found in online repositories. The names of the repository/repositories and accession number(s) can be found in the article/Supplementary Material.

## Author Contributions

VF-J and XL-A conducted all experiments with the help of AF-C, PE, AB-F, GR-M, and RG in the various parameters measured in the study. VF-J and NA led the writing of the MS. NA is the supervisor of the laboratory. All authors contributed to the article and approved the submitted version.

## Conflict of Interest

The authors declare that the research was conducted in the absence of any commercial or financial relationships that could be construed as a potential conflict of interest.
