# Supplementary material for: “The Good, the Bad and the Double-Sword” Effects of Microplastics and Their Organic Additives in Marine Bacteria
Source: Front Microbiol. 2021 Jan 20;11:581118. doi: 10.3389/fmicb.2020.581118 (PMC7854915; doi:10.3389/fmicb.2020.581118)
Supplement: Supplementary file 1 [file Data_Sheet_1.pdf]

Supplementary Table 1. List of all treatments performed in this study, divided into two parts: (**I**) Response to MPs at environmentally relevant concentrations and (**II**) Response to MPs at environmentally concentrations vs high concentrations (the "worst-case" scenario). Abbreviations: PE, polyethylene; PP, polypropylene; PVC, polyvinyl chloride; Fluo, fluoranthene; HBCD, 1,2,5,6,9,10-hexabromocyclododecane; DEHP, dioctyl-phthalate; PS, polystyrene. Response variables of each experiment are indicated in the table.

**Experimental plan/treatments:**		**MPs (PE, PP and/or PVC)**	**Additive (A) (Fluoranthene, HBCD and/or DEHP)**	**MPs and A: PE+Fluo/PP+HBCD/ PVC+DEHP or all interacting**	**PS (beads)**
**I. Response to MPs at environmentally relevant concentrations**					
Response variable: growth and microscopic analysis					
Species tested					
Phototroph: unicellular autotroph	*Halothece* sp. PCC 7418<br>Source: Pasteur Culture Collection	0, 0.01, 0.1, 1, 100 µg mL^{-1}	0, 0.3, 3, 30, 300 µg L^{-1}	Low (L) MPs (0.01 µg ml^{-1}) and Low (L) A (0.3 µg L^{-1})<br><br>High (H) MPs (100 µg ml^{-1}) and High (H) A (300 µg L^{-1})	4.55 x10^3 particles mL^{-1}<br><br>In *Halothece* sp. PCC 7418 (data did not show)
Phototroph: filamentous heterocyst-forming mixotroph	*Fischerella muscicola* PCC 73103<br>Source: Pasteur Culture Collection				
Heterotroph: isolated from roots of *P. oceanica*, benthic specie	*Cobetia* sp. UIB 001<br>Source: isolated directly from *P. oceanica* roots (Fernández-Juárez et al., in prep). GenBank: CP058244-CP058245				
Heterotroph: planktonic species	*Marinobacterium litorale* DSM 23545<br>Source: German Collection of Microorganisms and Cell Cultures GmbH (DSMZ)				
Heterotroph: planktonic species	*Pseudomonas azotifigens* DSM 17556^T:<br>Source: German Collection of Microorganisms and Cell Cultures GmbH (DSMZ)				
**II. Response to MPs at environmentally relevant concentrations vs high concentrations (the "worst-case" scenario)**					
Response variable: growth, microscopic analysis, protein overexpression, alkaline phosphatase activity (APA), PO_4^{3-}-uptake and N_2-fixation					
Species tested					
Phototroph: unicellular autotroph	*Halothece* sp. PCC 7418	0, 100 and 1000 µg mL^{-1}	0, 300, 3000 µg L^{-1}	3MPs (100 µg mL^{-1}) and 3A (300 µg L^{-1}) (all combined)<br><br>3MPs (1000 µg mL^{-1}) and 3A (3000 µg L^{-1}) (all combined)	4.55 x10^6 particles mL^{-1}<br><br>4.55 x10^7 particles mL^{-1}
Heterotroph: isolated from roots of *P. oceanica*, benthic specie	*Cobetia* sp. UIB 001				

Supplementary Table 2. Comparison of PO_4^{3-}-uptake (pmol PO_4^{3-} $cell^{-1}$ d^{-1}) between treatments using a posthoc test (Wilcoxon) after Kruskal-Wallis over the whole dataset in *Halothece* sp. and *Cobetia* sp. (significant differences = $p < 0.05$).

Halothece sp. (p-value)	Control	PS	PS^	PE	PE^	PP	PP	PVC	PVC^	Fluo	Fluo^	Br	Br^	DEHP	DEHP^	3MP	3MP	3A	3A^
Control	-	-	-	-	-	-	-	-	-	-	-	-	-	-	-	-	-	-	-
PS	0.08	-	-	-	-	-	-	-	-	-	-	-	-	-	-	-	-	-	-
PS^	0.02	0.80	-	-	-	-	-	-	-	-	-	-	-	-	-	-	-	-	-
PE	0.03	0.16	0.17	-	-	-	-	-	-	-	-	-	-	-	-	-	-	-	-
PE^	0.02	0.09	0.10	0.64	-	-	-	-	-	-	-	-	-	-	-	-	-	-	-
PP	0.02	0.40	0.44	0.37	0.20	-	-	-	-	-	-	-	-	-	-	-	-	-	-
PP^	0.00	0.38	0.36	0.33	0.18	0.95	-	-	-	-	-	-	-	-	-	-	-	-	-
PVC	0.09	0.47	0.54	0.59	0.37	0.88	0.85	-	-	-	-	-	-	-	-	-	-	-	-
PVC^	0.00	0.57	0.66	0.23	0.14	0.46	0.42	0.66	-	-	-	-	-	-	-	-	-	-	-
Fluo	0.09	0.24	0.27	0.71	0.14	0.39	0.38	0.48	0.32	-	-	-	-	-	-	-	-	-	-
Fluo^	0.04	0.09	0.27	0.21	0.94	0.13	0.14	0.14	0.12	0.39	-	-	-	-	-	-	-	-	-
HBCD	0.14	0.56	0.31	0.07	0.30	0.13	0.09	0.27	0.16	0.17	0.07	-	-	-	-	-	-	-	-
HBCD^	0.04	0.25	0.08	0.05	0.04	0.04	0.01	0.17	0.01	0.14	0.07	0.37	-	-	-	-	-	-	-
DEHP	0.09	0.88	0.66	0.13	0.08	0.31	0.29	0.40	0.44	0.22	0.08	0.67	0.29	-	-	-	-	-	-
DEHP^	0.30	0.95	0.95	0.42	0.30	0.71	0.83	0.67	0.84	0.34	0.12	0.72	0.52	0.88	-	-	-	-	-
3MP	0.28	0.54	0.29	0.07	0.05	0.12	0.09	0.26	0.15	0.17	0.07	0.96	0.40	0.64	0.71	-	-	-	-
3MP^	0.21	0.20	0.04	0.04	0.03	0.03	0.00	0.12	0.00	0.11	0.06	0.16	0.25	0.16	0.40	0.18	-	-	-
3A	0.51	0.07	0.05	0.02	0.02	0.03	0.07	0.06	0.08	0.06	0.03	0.10	0.20	0.07	0.22	0.10	0.29	-	-
3A^	0.03	0.19	0.22	0.63	0.86	0.32	0.31	0.41	0.26	0.94	0.41	0.14	0.11	0.17	0.30	0.13	0.09	0.04	-

Cobetia sp. (p-value)	Control	PS	PS^	PE	PE	PP	PP^	PVC	PVC^	Fluo	Fluo^	Br	Br^	DEHP	DEHP^	3MP	3MP^	3A	3A^
Control	-	-	-	-	-	-	-	-	-	-	-	-	-	-	-	-	-	-	-
PS	0.08	-	-	-	-	-	-	-	-	-	-	-	-	-	-	-	-	-	-
PS^	0.01	0.00	-	-	-	-	-	-	-	-	-	-	-	-	-	-	-	-	-
PE	0.48	0.19	0.01	-	-	-	-	-	-	-	-	-	-	-	-	-	-	-	-
PE^	0.66	0.21	0.03	0.89	-	-	-	-	-	-	-	-	-	-	-	-	-	-	-
PP	0.00	0.39	0.00	0.34	0.36	-	-	-	-	-	-	-	-	-	-	-	-	-	-
PP^	0.13	0.07	0.01	0.27	0.41	0.00	-	-	-	-	-	-	-	-	-	-	-	-	-
PVC	0.26	0.24	0.01	0.74	0.67	0.47	0.14	-	-	-	-	-	-	-	-	-	-	-	-
PVC^	0.04	0.90	0.00	0.17	0.20	0.77	0.02	0.22	-	-	-	-	-	-	-	-	-	-	-
Fluo	0.13	0.23	0.00	0.62	0.20	0.48	0.07	0.87	0.20	-	-	-	-	-	-	-	-	-	-
Fluo^	0.01	0.01	0.00	0.01	0.57	0.00	0.01	0.00	0.00	0.00	-	-	-	-	-	-	-	-	-
HBCD	0.35	0.03	0.01	0.30	0.02	0.02	0.89	0.15	0.02	0.07	0.00	-	-	-	-	-	-	-	-
HBCD^	0.02	0.02	0.02	0.10	0.17	0.00	0.07	0.04	0.01	0.02	0.00	0.13	-	-	-	-	-	-	-
DEHP	0.30	0.04	0.10	0.19	0.26	0.08	0.50	0.12	0.04	0.10	0.03	0.48	0.87	-	-	-	-	-	-
DEHP^	0.01	0.06	0.05	0.09	0.14	0.00	0.03	0.05	0.01	0.03	0.04	0.08	0.40	0.64	-	-	-	-	-
3MP	0.01	0.04	0.03	0.10	0.17	0.00	0.02	0.05	0.01	0.02	0.01	0.11	0.91	0.83	0.28	-	-	-	-
3MP^	0.55	0.05	0.03	0.33	0.45	0.09	0.95	0.19	0.04	0.14	0.01	0.90	0.37	0.59	0.25	0.35	-	-	-
3A	0.04	0.01	0.09	0.05	0.09	0.01	0.06	0.02	0.00	0.01	0.01	0.06	0.29	0.45	0.50	0.30	0.16	-	-
3A^	0.07	0.29	0.00	0.08	0.10	0.17	0.07	0.09	0.33	0.08	0.01	0.03	0.03	0.03	0.06	0.05	0.06	0.01	-

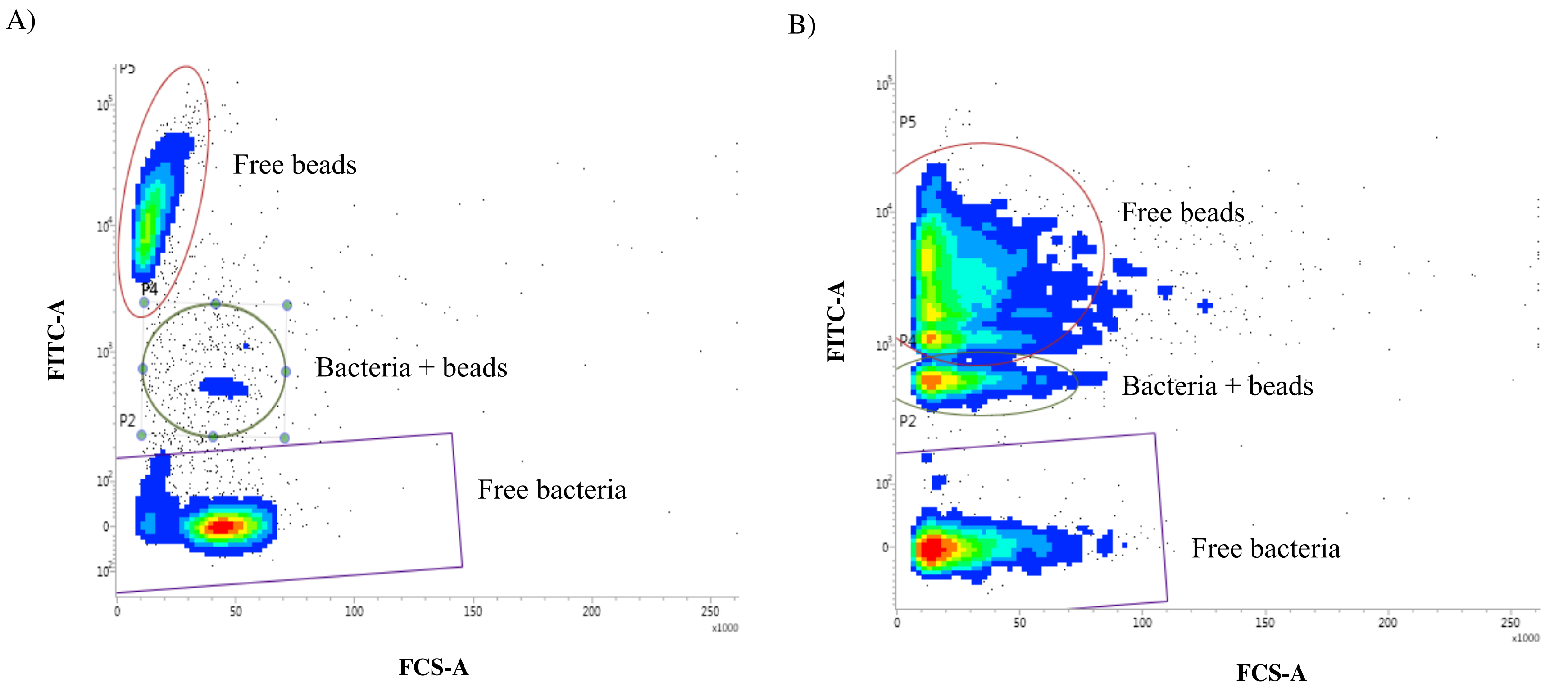

Supplementary Figure 1. Cytograms showing **A)** *Halothece* sp. and **B)** *Cobetia* sp. cells, being free or adsorbed with the PS-beads, using FITC-A and FCS-A as the flow cytometer parameters.
